# The 2D:4D Digit Ratio and Primary Sjögren's Syndrome: A Window Into Prenatal Hormonal Influences on Exocrine Autoimmunity

**DOI:** 10.1002/ajhb.70257

**Published:** 2026-04-09

**Authors:** Mustafa Gür, Ramazan Fazil Akkoc, Burak Oz, Ahmet Karatas, Süleyman Serdar Koca

**Affiliations:** ^1^ Department of Rheumatology, Faculty of Medicine Firat University Elazig Turkey; ^2^ Department of Anatomy, Faculty of Medicine Firat University Elazig Turkey

**Keywords:** 2D:4D, autoimmune disease, prenatal androgen, Sjögren's syndrome

## Abstract

**Objectives:**

The pronounced female preponderance in Sjögren's syndrome (SS) points to a potential role for sex hormones in disease development. The ratio of the second to fourth digit (2D:4D), which serves as an indirect marker of fetal androgen exposure, has shown associations with several immune‐mediated conditions. The present study assessed whether 2D:4D ratios are lower in women diagnosed with SS relative to healthy individuals.

**Methods:**

In this case–control investigation, 57 female SS patients (classified per the 2016 ACR/EULAR criteria) and 44 age‐matched healthy women were recruited. The lengths of the index and ring fingers were measured on both hands with digital calipers, and digit ratios were derived. Group comparisons were conducted with Welch's *t*‐test and supplemented by Mann–Whitney *U*‐tests.

**Results:**

Women with SS had lower 2D:4D ratios on both sides (right: 0.938 ± 0.030 vs. 1.011 ± 0.024, *p* < 0.001; left: 0.925 ± 0.034 vs. 1.009 ± 0.026, *p* < 0.001). The observed effect sizes were large (Cohen's *d* > 2.6). Lower ratios were driven predominantly by elongated ring fingers rather than shortened index fingers, a pattern compatible with heightened fetal androgen exposure.

**Conclusions:**

Women with primary SS exhibit lower 2D:4D digit ratios compared with healthy controls, suggesting that a shifted prenatal estrogen‐to‐androgen balance may prime the immune system toward the glandular autoimmunity and exocrine dysfunction that define this disease. These findings implicate the prenatal hormonal milieu as a potential contributor to the immunological processes underlying SS pathogenesis.

## 1 Introduction

Sjögren's syndrome (SS) is a chronic systemic autoimmune disorder in which lymphocytic infiltration targets the exocrine glands, primarily the salivary and lacrimal glands, producing the hallmark symptoms of oral and ocular dryness. Epidemiologically, SS is characterized by a conspicuous sex imbalance, with women representing roughly 90% of affected individuals and reported female‐to‐male ratios between 9:1 and 20:1 (Brito‐Zerón et al. 2016; Fox 2005). Such a pronounced disparity suggests that hormonal factors play a role in the disease process.

A substantial body of evidence supports the involvement of sex steroids in autoimmune disease susceptibility beyond what epidemiological sex ratios alone convey. Androgens exert critical trophic and regulatory effects on exocrine glands; the TFOS DEWS II Sex, Gender, and Hormones Report documented that androgen deficiency contributes to meibomian gland dysfunction and lacrimal gland inflammation, both of which are central features of SS (Sullivan et al. 2017). Moreover, fluctuations in sex hormone levels during pregnancy, menopause, and hormonal therapy are associated with disease onset or flare in multiple autoimmune conditions, including SS (Brito‐Zerón et al. 2016). These observations collectively provide a strong rationale for investigating markers of the prenatal hormonal environment in the context of SS susceptibility.

One anthropometric index that has attracted considerable research interest as an indirect gauge of the prenatal hormonal environment is the 2D:4D ratio, obtained by dividing the length of the index finger by that of the ring finger. This ratio is thought to mirror the balance between testosterone and estrogen during the embryonic period (Manning and Fink 2018; Yan et al. 2023). On average, men have lower values than women, a dimorphism that is established in utero and remains stable across the lifespan (Galis et al. 2010; Kumar et al. 2017; Pruszkowska‐Przybylska et al. 2024). Richards et al. (2022) confirmed meaningful correlations between circulating maternal hormones in early gestation and the digit ratios of offspring, while the molecular basis appears to involve region‐specific androgen receptor density in the developing finger buds (Zheng and Cohn 2011; Auger et al. 2013).

A growing literature has explored the clinical relevance of digit ratio across a wide range of disorders. Jeevanandam and Muthu (2016) summarized links with cardiovascular pathology, certain cancers, and autoimmune diseases. Among immune‐mediated conditions, Święchowicz et al. (2022) documented atypical ratios in women with autoimmune thyroid diseases, including Hashimoto's thyroiditis and Graves' disease. Bove et al. (2015) found that men with multiple sclerosis differed from healthy counterparts in their digit ratios, and Uğur et al. (2019) examined the potential utility of 2D:4D as a marker of disease activity in ankylosing spondylitis. Of particular relevance, Akkoc et al. (2026) recently reported that female patients with systemic sclerosis (SSc) display substantially lower digit ratios than healthy women, offering the first such evidence in a fibrotic autoimmune condition.

The sex difference in digit ratio is remarkably robust, being detectable from the fetal period onward and replicated across diverse ethnic populations (Galis et al. 2010; Kumar et al. 2019; Ernsten et al. 2021). Animal experiments have further shown that in utero exposure to estrogenic or antiandrogenic agents can shift the ratio, underscoring the measure's sensitivity to perturbations of the prenatal hormonal environment (Auger et al. 2013; Redmond and Ash 2017).

In light of the pronounced sex bias observed in SS and the recent digit ratio findings in SSc, we hypothesized that women with SS would exhibit lower 2D:4D digit ratios than unaffected women, consistent with higher prenatal androgen exposure. To the best of our knowledge, no published investigation has assessed the 2D:4D ratio in patients with SS. The aim of the present study was to evaluate whether female SS patients show lower digit ratios compared with healthy controls, which could imply a contribution of the prenatal androgenic environment to SS susceptibility.

## 2 Materials and Methods

### 2.1 Study Design and Participants

A case–control design was employed at the Rheumatology Department of Fırat University Faculty of Medicine. A total of 57 women fulfilling the 2016 ACR/EULAR classification criteria for primary SS formed the patient cohort. A total of 44 age‐matched healthy women who had no documented autoimmune, connective tissue, or endocrine disorders served as controls.

### 2.2 Anthropometric Measurements

Stature and body weight were obtained with conventional clinical instruments. Body mass index (BMI) was derived by dividing weight (kg) by the square of height (m).

### 2.3 Digit Length Measurements

**FIGURE 1:**
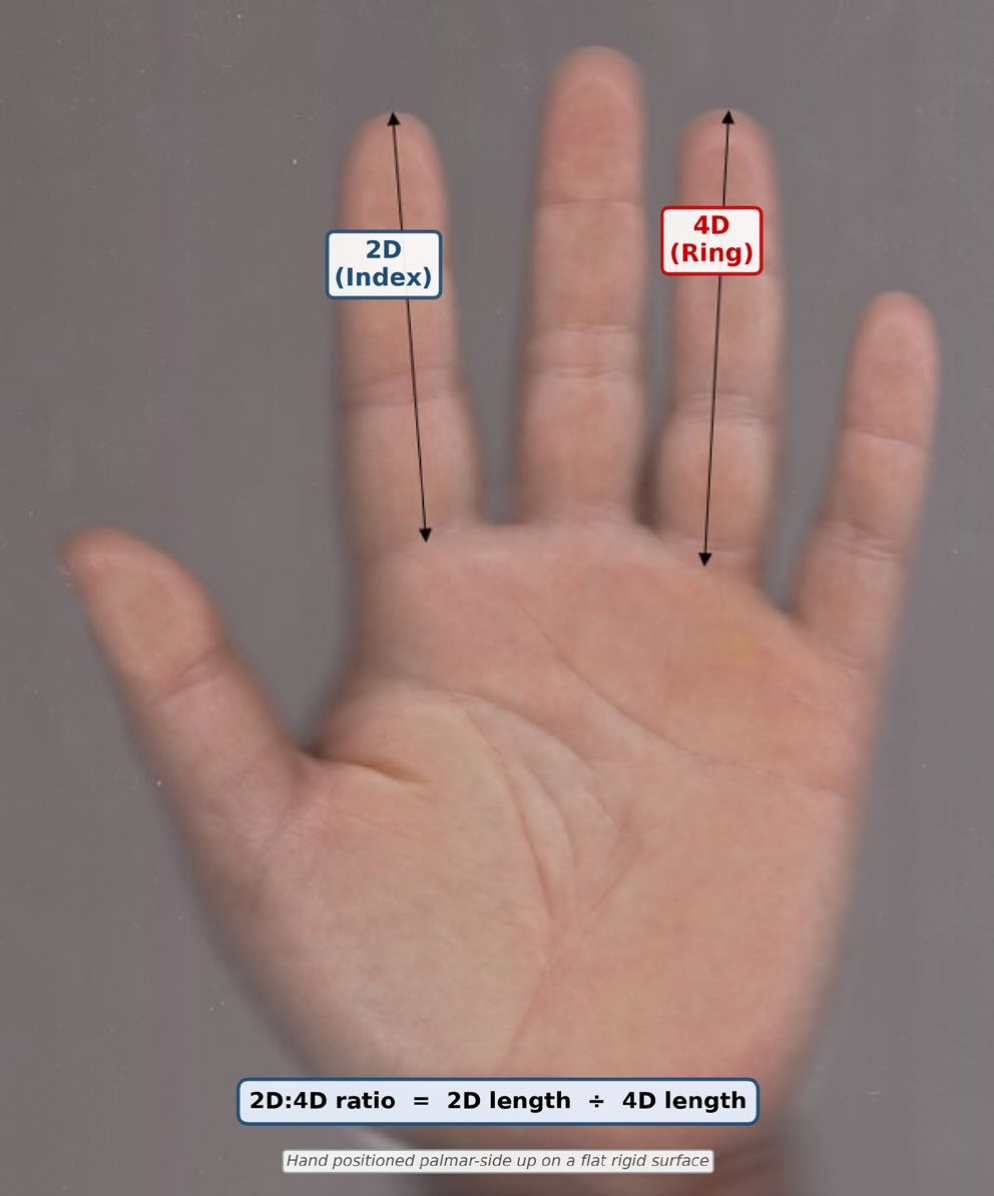
Schematic illustration of the digit measurement technique. The participant's hand is placed palmar‐side up on a flat surface. Calibrated digital vernier calipers are positioned to measure the distance from the most proximal palmar crease of each digit to the fingertip. Measurements of the second (index) and fourth (ring) fingers are shown.

For digit measurement, each participant placed both hands dorsal‐side down on a rigid flat surface with the palmar aspect facing upward. The thumb was gently abducted while the remaining four fingers were held in an adducted position. Using calibrated digital vernier calipers (Servi et al. 2025; Akkoc et al. 2026), the distance from the most proximal palmar crease of the second and fourth digits to the respective fingertip was measured on the palmar surface. All measurements were performed by a single experienced anatomist (R.F.A.) to eliminate interobserver variability. The investigator measured each participant twice; however, the individual repeated measurement values were not recorded separately, precluding formal intraclass correlation coefficient (ICC) computation. The measurement technique is illustrated in Figure 1.

### 2.4 Statistical Analysis

All analyses were carried out in SPSS 26.0 (IBM Corp., Armonk, NY, USA). Continuous data are reported as mean ± standard deviation along with median and interquartile range when appropriate. The Shapiro–Wilk test was used to evaluate distributional normality and Levene's test to check variance equality. Because group variances were unequal for several variables, Welch's *t*‐test served as the primary parametric comparison; Mann–Whitney *U*‐tests provided nonparametric confirmation. Effect magnitude was quantified with Cohen's *d*. The discriminative capacity of digit ratios was appraised through receiver operating characteristic (ROC) curves and the corresponding areas under the curve (AUC). Bivariate associations were assessed with Pearson's correlation coefficient. The laterality index (D*r‐l*) was computed as the difference between right‐hand and left‐hand 2D:4D for each participant and compared between groups. To address potential allometric effects of body size on digit lengths, an analysis of covariance (ANCOVA) was performed with height as a covariate. A post hoc power analysis was conducted using G*Power 3.1 (Faul et al. 2007). Two‐tailed *p* values below 0.05 were deemed statistically significant.

## 3 Results

### 3.1 Participant Characteristics

A total of 101 women participated: 57 with SS and 44 healthy controls. Table 1 summarizes the demographic and anthropometric data. No statistically significant differences emerged between the two groups for age (SS: 49.2 ± 12.0 years; controls: 46.2 ± 13.7 years; *p* = 0.259), BMI (SS: 26.3 ± 5.4 kg/m^2^; controls: 25.8 ± 3.1 kg/m^2^; *p* = 0.581), stature, or body weight, confirming adequate matching.

**TABLE 1:** Demographic and anthropometric characteristics of study groups.

Variable	SS (*n* = 57)	Control (*n* = 44)	*p*
Age (years)	49.16 ± 11.95	46.20 ± 13.67	0.259
Height (cm)	160.07 ± 8.32	160.68 ± 6.80	0.685
Weight (kg)	67.33 ± 14.99	66.34 ± 7.09	0.661
BMI (kg/m^2^)	26.26 ± 5.44	25.79 ± 3.09	0.581
Female sex, *n* (%)	57 (100)	44 (100)	—

*Note:* Data are presented as mean ± standard deviation or *n* (%).

Abbreviations: BMI, body mass index; SS, Sjögren's syndrome.

### 3.2 2D:4D Ratio Comparisons

**FIGURE 2:**
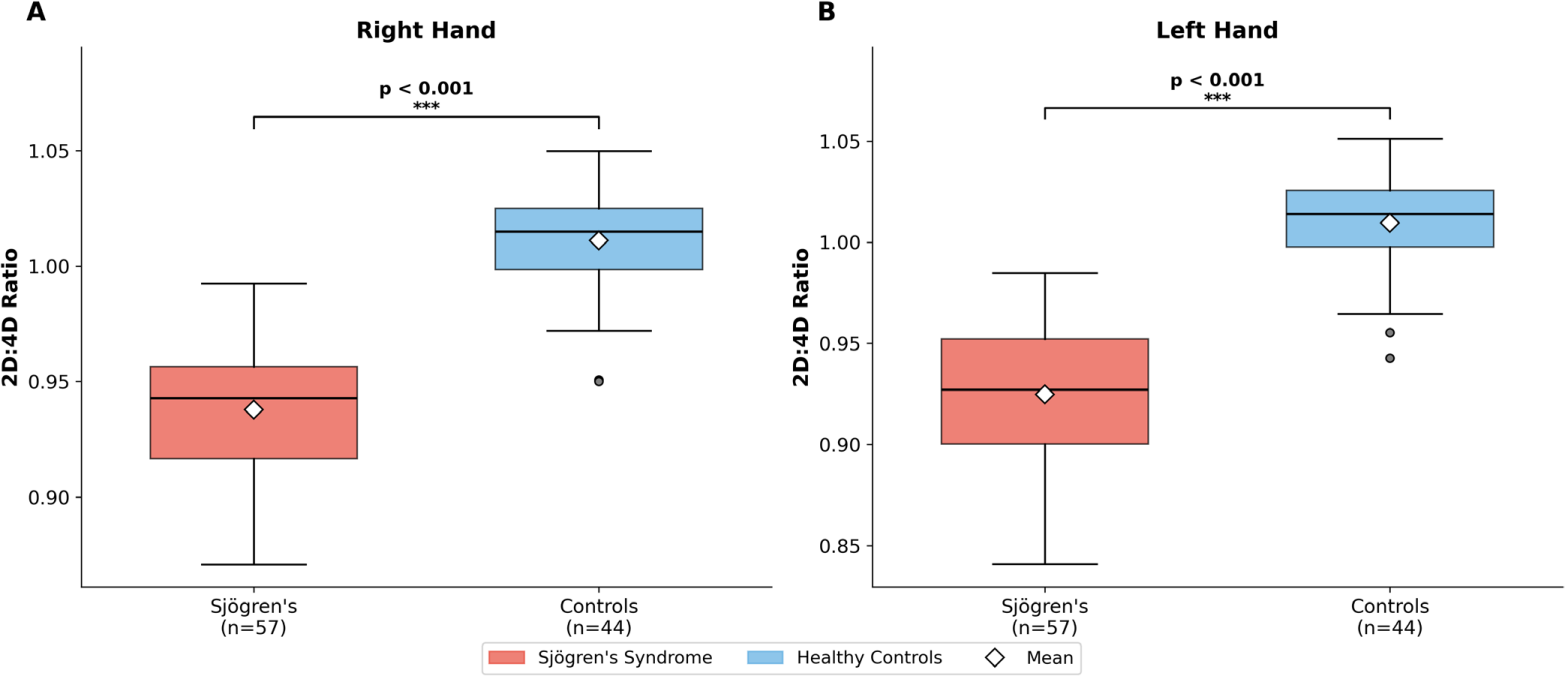
Box plots comparing 2D:4D digit ratios between women with Sjögren's Syndrome and healthy controls. (A) Right hand. (B) Left hand. Horizontal lines indicate medians, diamond markers indicate means, boxes span the interquartile range, whiskers extend to 1.5× IQR, and circles denote outliers. ***p < 0.001.

Digit ratio data are presented in Table 2 and illustrated in Figure 2. Women with SS showed consistently lower 2D:4D values than controls on both sides. The right‐hand ratio averaged 0.938 ± 0.030 in patients and 1.011 ± 0.024 in controls (*t* = −13.56, *p* < 0.001); the left‐hand ratio was 0.925 ± 0.034 versus 1.009 ± 0.026 (*t* = −14.26, *p* < 0.001). Mean between‐group differences amounted to approximately 0.073 on the right and 0.085 on the left. In neither case did the 95% confidence interval encompass zero (right: −0.084 to −0.063; left: −0.097 to −0.073).

**TABLE 2:** Comparison of 2D:4D ratios, digit lengths, and laterality index (D*r‐l*) between groups.

Variable	SS (*n* = 57)	Control (*n* = 44)	*p*
Right hand			
2D:4D ratio	0.938 ± 0.030	1.011 ± 0.024	**< 0.001**
2D length (cm)	6.716 ± 0.452	6.824 ± 0.409	0.209
4D length (cm)	7.165 ± 0.489	6.752 ± 0.416	**< 0.001**
Left hand			
2D:4D ratio	0.925 ± 0.034	1.009 ± 0.026	**< 0.001**
2D length (cm)	6.568 ± 0.471	6.823 ± 0.435	**0.006**
4D length (cm)	7.106 ± 0.489	6.761 ± 0.431	**< 0.001**
Laterality			
D*r‐l*	0.012 ± 0.030	−0.001 ± 0.020	**0.014**

*Note:* Data are presented as mean ± standard deviation. Bold *p* values indicate statistical significance (*p* < 0.05).

Abbreviations: 2D, second digit; 4D, fourth digit; D*r‐l*, right minus left 2D:4D ratio (laterality index); SS, Sjögren's syndrome.

The magnitude of group separation was large: Cohen's *d* exceeded −2.6 bilaterally, substantially surpassing the conventional large‐effect benchmark of 0.8. The post hoc power analysis confirmed that the study had > 99% power to detect these effect sizes at *α* = 0.05 with the present sample. The laterality index (D*r‐l* = right minus left 2D:4D) differed significantly between groups (SS: 0.012 ± 0.030 vs. controls: −0.001 ± 0.020; *t* = 2.52, *p* = 0.014; Cohen's *d* = 0.49), indicating that women with SS had relatively higher right‐hand than left‐hand 2D:4D ratios compared with controls.

### 3.3 Individual Digit Measurements

**FIGURE 3:**
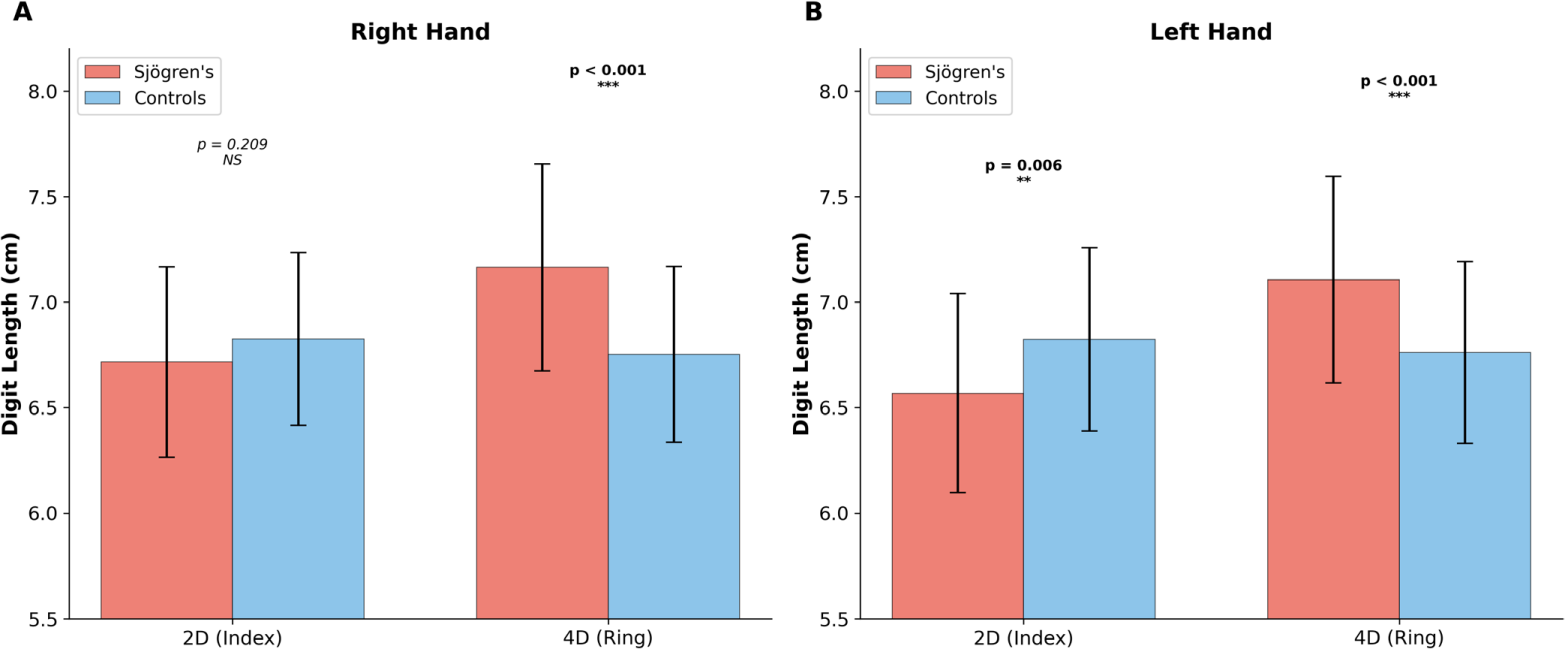
Comparison of index (2D) and ring (4D) finger lengths in patients with Sjögren's syndrome and healthy controls. (A) Right hand. (B) Left hand. Bars represent means; error bars indicate standard deviations. NS, not significant; **p < 0.01; ***p < 0.001.

When individual digit lengths were examined (Figure 3), index finger length did not differ significantly between groups on the right side (6.72 vs. 6.82 cm, *p* = 0.209). However, on the left side, patients had shorter index fingers (6.57 vs. 6.82 cm, *p* = 0.006). Ring finger length was significantly greater in SS patients on both hands (right: 7.17 vs. 6.75 cm, *p* < 0.001; left: 7.11 vs. 6.76 cm, *p* < 0.001). Thus, the lower digit ratios in SS stem chiefly from elongated ring fingers, supplemented on the left by shorter index fingers.

When height was included as a covariate in ANCOVA, the group differences in 2D:4D remained highly significant for both the right hand (*F* = 149.43, *p* < 0.001) and the left hand (*F* = 185.49, *p* < 0.001), indicating that the observed ratio differences are not attributable to allometric scaling effects. The covariate (height) was not significant in either model (right: *F* = 1.64, *p* = 0.203; left: *F* = 0.15, *p* = 0.704). This finding is further supported by the absence of a significant height difference between the groups (*p* = 0.685).

### 3.4 ROC Analysis

**FIGURE 4:**
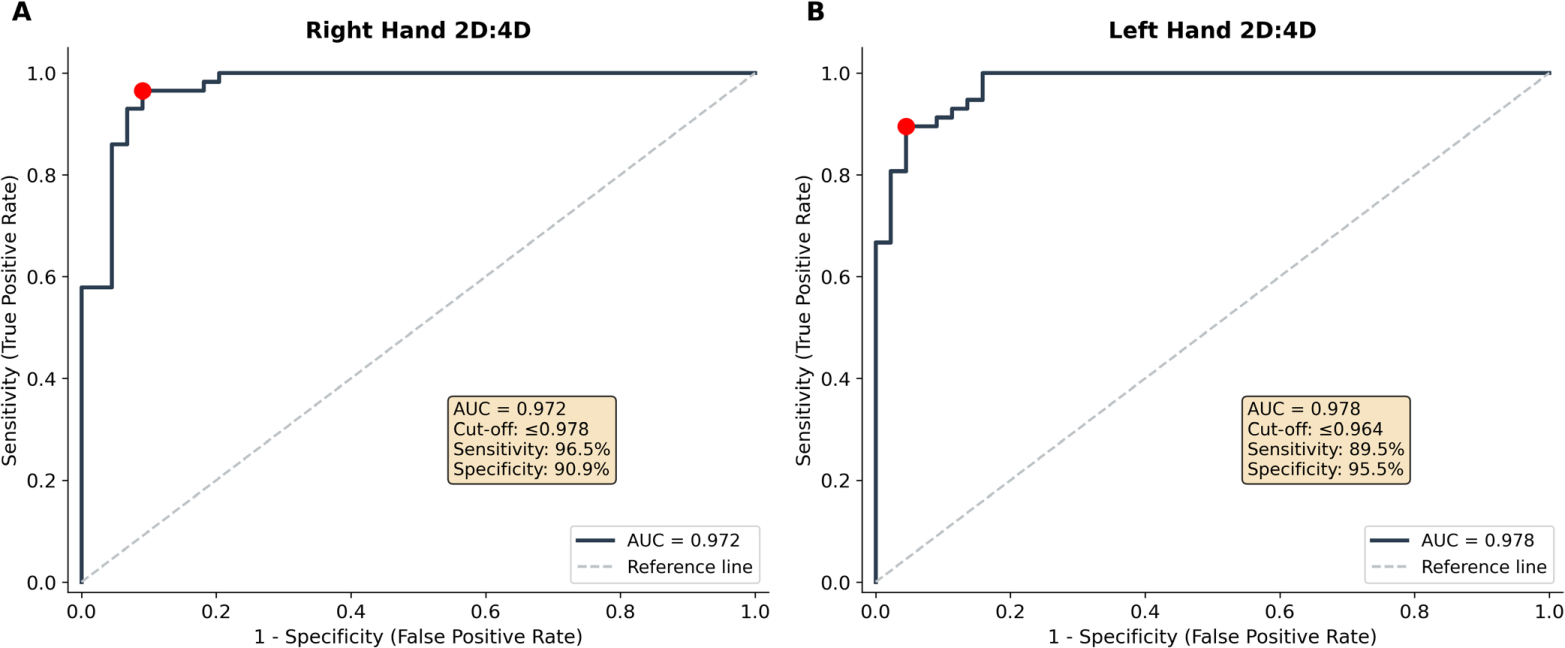
ROC curve analysis evaluating the discriminative performance of the 2D:4D ratio for Sjögren's syndrome. (A) Right hand. (B) Left hand. The red dot indicates the optimal cut‐off point determined by the Youden index.

ROC curve analysis (Figure 4) yielded AUC values of 0.972 (right hand) and 0.978 (left hand). The Youden index–derived optimal threshold for the right hand was ≤ 0.978, corresponding to 96.5% sensitivity and 90.9% specificity; for the left hand, the optimal cutoff was ≤ 0.964, with 89.5% sensitivity and 95.5% specificity.

### 3.5 Correlation Analyses

In the SS cohort, digit ratio showed no meaningful linear association with age (right: *r* = −0.21, *p* = 0.113; left: *r* = −0.20, *p* = 0.140) or BMI (right: *r* = −0.15, *p* = 0.252; left: *r* = −0.20, *p* = 0.143). A strong positive correlation between right‐ and left‐hand ratios was present in both SS patients (*r* = 0.58, *p* < 0.001) and controls (*r* = 0.82, *p* < 0.001), in line with reports from other populations (Kaneoke et al. 2017; Kowal et al. 2020).

## 4 Discussion

The central observation of this study is that women diagnosed with SS have lower 2D:4D ratios than their healthy counterparts. Because this ratio is regarded as a proxy for the prenatal testosterone‐to‐estrogen balance, the results imply that SS patients were exposed to comparatively higher androgen levels during embryonic life.

This observation extends and corroborates a recent report on SSc in which a similar pattern of lower digit ratios was observed in female SSc patients (Akkoc et al. 2026). That the same pattern emerges in two mechanistically distinct autoimmune rheumatic diseases, one dominated by fibrosis and vasculopathy and the other by glandular lymphocytic infiltration, raises the possibility that elevated prenatal androgen exposure constitutes a common predisposing factor for autoimmune connective tissue disorders rather than being tied to a single pathogenic mechanism.

At first glance, an association between higher fetal testosterone and a disease that predominantly affects women may seem counterintuitive. Nonetheless, the interplay between prenatal androgens and immune ontogeny is multifaceted. Hormonal signals received in utero can durably reprogram the developing immune system, shaping lymphocyte repertoires and tolerance checkpoints in ways that may only become clinically apparent decades later (Evardone and Alexander 2009). Androgens are well recognized for their capacity to modulate both innate and adaptive immunity, and fetal exposure may establish immune set points that favor autoimmune reactivity under subsequent environmental triggers.

Our findings converge with those reported in other autoimmune contexts. Święchowicz et al. (2022) observed atypical digit ratios in autoimmune thyroid disease, and Bove et al. (2015) documented analogous differences in male multiple sclerosis patients. Across these diverse conditions, the recurrent theme is that digit ratios diverge from those of healthy populations, implicating the fetal hormonal environment as a shared contributor to autoimmune vulnerability.

Dissection of individual digit lengths showed that the lower ratio in SS was driven predominantly by elongated ring fingers, which is biologically coherent given the higher density of androgen receptors in fourth‐digit primordia relative to second‐digit primordia (Zheng and Cohn 2011). An additional observation distinguishing SS from SSc was the significantly shorter left‐hand index finger in SS patients (*p* = 0.006), indicating that both augmented fourth digit and attenuated second‐digit growth may jointly account for the lower left‐hand ratio. This lateralization pattern merits further exploration.

Multiple gestational factors, maternal psychological stress, nutritional status, environmental endocrine disruptors, and co‐twin sex are known to modulate fetal testosterone concentrations (Auger et al. 2013; Kasielska‐Trojan et al. 2022). Whether any of these exposures simultaneously raise testosterone levels and confer vulnerability to SS in later life remains an open question.

How prenatal androgens could predispose to a disease that manifests decades later is necessarily speculative but not without biological plausibility. In the specific context of SS, androgens exert important trophic and protective effects on salivary and lacrimal gland tissue; androgen insufficiency has been linked to meibomian gland atrophy and aqueous‐deficient dry eye (Sullivan et al. 2017). One may speculate that fetal androgen programming shapes exocrine gland development in ways that render these structures more susceptible to immune‐mediated destruction in adulthood. In parallel, prenatal hormonal conditioning of immune cell lineages may shift the equilibrium between self‐tolerance and autoreactivity. Pruszkowska‐Przybylska et al. (2021) showed that digit ratio correlates with a range of metabolic and endocrine parameters, underscoring the far‐reaching physiological consequences of the prenatal hormonal milieu.

An alternative or complementary interpretation invokes genetic pleiotropy (Cotsapas et al. 2011). HOX‐family genes that govern digit patterning during embryogenesis (Zheng and Cohn 2011) also appear to participate in immune system maturation (Rux and Wellik 2017). Certain allelic variants might therefore simultaneously determine digit ratios and immune phenotypes, producing a statistical association without a direct hormonal pathway. Equally, polymorphisms in androgen receptor or related signaling genes that are enriched among SS patients could influence fourth‐digit growth independently of systemic hormone concentrations (Manning et al. 2013). Under either scenario, the digit ratio would function as a developmental biomarker of genetic risk rather than a straightforward readout of circulating fetal testosterone. These hypotheses are not mutually exclusive, and their resolution will likely require genome‐wide association studies combined with detailed morphometric phenotyping.

The significant group difference in the laterality index (D*r‐l*; *p* = 0.013) contrasts with our SSc data, where no such asymmetry was detected (Akkoc et al. 2026). The mean D*r‐l* was 0.012 ± 0.030 in the SS group and −0.001 ± 0.020 in controls (Cohen's *d* = 0.49), indicating that women with SS showed a greater rightward asymmetry in digit ratio. This directional asymmetry, in which the right‐hand ratio exceeds the left, may reflect lateralized differences in sensitivity to prenatal androgens. Laterality in digit ratio has been proposed to reflect developmental instability and may carry information about the intrauterine environment that is independent of overall ratio magnitude (Kasielska‐Trojan et al. 2022). The discrepancy between SS and SSc in this regard warrants a dedicated investigation.

The question of whether body size exerts an allometric influence on digit ratio has been debated in the 2D:4D literature. In our study, the two groups were well matched for height (*p* = 0.685), and ANCOVA with height as a covariate did not alter the significance of the group differences (right: *F* = 149.43, *p* < 0.001; left: *F* = 185.49, *p* < 0.001), while height itself was not a significant covariate. These findings suggest that the observed ratio differences reflect genuine biological variation in digit proportions rather than a scaling artifact.

Regarding measurement methodology, it should be noted that indirect techniques such as photocopies, radiographs, or digital photographs analyzed with image‐processing software have been reported to offer higher precision than direct caliper measurements in some studies. We chose direct measurement because of its practicality in a clinical setting and its established precedent in the 2D:4D literature, including recent reports using comparable protocols (Servi et al. 2025; Akkoc et al. 2026). The fact that highly significant between‐group differences were detected despite any additional measurement variability inherent to the direct approach suggests that the biological signal was robust.

Several limitations should be acknowledged. First, although the same investigator performed all measurements and repeated each measurement twice, the individual repeated values were not recorded separately, which precluded formal ICC computation. Future studies should incorporate a prospectively designed reliability protocol with separately recorded duplicate measurements. Second, although the sample size was sufficient to detect the large effect sizes observed, it precluded stratified analyses by serological subtype (anti‐Ro/La status) or extraglandular manifestations. Post hoc power analysis confirmed > 99% power for the observed effects; however, as with any single‐center study, the large effect sizes could partly reflect sampling variability, and replication in independent cohorts is essential before definitive conclusions about effect magnitude can be drawn. We did not measure circulating hormones or other surrogates of prenatal exposure. The cross‐sectional design precludes causal inference, and only women were included, restricting generalizability. Furthermore, the comparison between SS and SSc results, although informative, derives from a single center's control pool; multicenter replication is essential.

Notwithstanding these caveats, the effect sizes observed here (Cohen's *d* > 2.6) are among the largest reported in 2D:4D studies of autoimmune diseases. For context, Akkoc et al. (2026) reported effect sizes of comparable magnitude in SSc, while Święchowicz et al. (2022) observed smaller but significant differences in autoimmune thyroid disease. The ROC analysis returned AUC values above 0.97, but these figures should be interpreted with restraint; they likely reflect the modest sample size and the particular demographic makeup of the cohort rather than genuine diagnostic potential. The 2D:4D ratio is not proposed as a clinical diagnostic tool for SS, and independent validation in larger and more heterogeneous populations is indispensable.

## 5 Conclusion

Female patients with SS exhibit lower 2D:4D ratios than healthy women, consistent with heightened prenatal testosterone exposure. Taken together with analogous observations in SSc, these data strengthen the evidence that the intrauterine hormonal environment may help shape susceptibility to autoimmune rheumatic diseases and contribute to the pronounced sex asymmetry characteristic of these conditions.

## Author Contributions

**Mustafa Gür:** conceptualization, resources, investigation, writing – review and editing. **Ramazan Fazil Akkoc:** methodology, data curation, formal analysis, writing – original draft, writing – review and editing. **Burak Oz:** investigation, data curation, writing – review and editing. **Ahmet Karatas:** investigation, writing – review and editing, supervision. **Süleyman Serdar Koca:** conceptualization, supervision, writing – review and editing, project administration.

## Funding

The authors have nothing to report.

## Ethics Statement

Approval for this noninterventional study was granted by the Firat University Non‐Interventional Research Ethics Committee (session number: 2025/09; decision number: 15; date: 03.07.2025). The study was carried out in compliance with the principles of the Declaration of Helsinki.

## Consent

Written informed consent was obtained from every participant prior to enrollment.

## Conflicts of Interest

The authors declare no conflicts of interest.

## Data Availability

The data that support the findings of this study are available from the corresponding author upon reasonable request.
